# Development of a novel fusion protein with *Anaplasma marginale* and *A*. *centrale* MSP5 improved performance of *Anaplasma* antibody detection by cELISA in infected and vaccinated cattle

**DOI:** 10.1371/journal.pone.0211149

**Published:** 2019-01-23

**Authors:** María E. Primo, Carolina S. Thompson, Beatriz S. Valentini, Macarena Sarli, María B. Novoa, Atilio J. Mangold, Susana T. de Echaide

**Affiliations:** 1 Instituto Nacional de Tecnología Agropecuaria, Estación Experimental Agropecuaria Rafaela, Rafaela, Santa Fe, Argentina; 2 Consejo Nacional de Investigaciones Científicas y Técnicas (CONICET), Santa Fe, Argentina; University of Minnesota, UNITED STATES

## Abstract

Detection of antibodies to *Anaplasma* spp. using commercial competitive enzyme-linked immunosorbent assay (ccELISA) is based on the recombinant major surface protein 5 fused to maltose binding protein (MBP-MSP5) or glutathione S-transferase (GST-MSP5). To avoid false positive reactions due to the presence of antibodies against *E*. *coli* MBP in cattle, previous sera absorption is required. This study evaluated the replacement of MBP-MSP5 or GST-MSP5 antigens by the truncate MSP5 (residues 28–210) of *A*. *marginale* (tMSP5m), *A*. *centrale* (tMSP5c) and fusion protein MSP5 (tMSP5cm), expressed without N-terminus transmembrane helix in the ccELISA test. Immunoreactivity was evaluated by western blot using monoclonal antibodies against the tMSP5 and by *in-house* cELISA (hcELISA) with purified tMSP5m, tMSP5c or tMSP5cm using sera from cattle infected with *A*. *marginale* (n = 226) or vaccinated with *A*. *centrale* (n = 173) and uninfected cattle (n = 216). Results of hcELISA were compared with those of ccELISA. Recombinant protein was expressed highly soluble (> 95%) in *E*. *coli* without a molecular chaperone. Specificity of the hcELISA-tMSP5m, -MSP5c or -tMSP5cm was identical to (99.5%) and greater than that in ccELISA (96.3%). Sensitivity of hcELISA-tMSP5m and ccELISA was identical (95.5%), but lower than that of hcELISA-tMSP5cm (96.2%) and -tMSP5c (97.2%). The analysis of vaccinated cattle by hcELISA-tMSP5c showed sensitivity of 99.4%. In summary, the generation of fusion MSP5 *A*. *marginale*-*A*. *centrale* protein without transmembrane helix was a very effective method to express the recombinant protein highly soluble in the bacterial cytoplasm and contributed to an increased test performance for detecting antibodies in cattle naturally infected with *A*. *marginale* or vaccinated with *A*. *centrale*.

## Introduction

Bovine anaplasmosis is an infectious disease caused by the obligate intraerythrocytic bacterium *Anaplasma marginale* [1] and is transmitted biologically to susceptible cattle by ticks or mechanically by biting flies and fomites [2–4]. The disease is endemic in mainly tropical and subtropical areas of the world. In Argentina, *A*. *marginale* is prevalent north of 33°S; nevertheless, anaplasmosis outbreaks have been detected to the south of the endemic zone due to the movement of carrier cattle to non-endemic areas [5,6]. Acute anaplasmosis affects mostly adult bovines and is characterized by severe anemia with destruction of erythrocytes, abortion, weight loss, reduced milk production and death. Cattle that recover from acute disease remain carriers and serve as reservoirs for transmission to other animals [7]. Immunization with live *A*. *centrale*, a species closely related to *A*. *marginale*, is used to prevent acute anaplasmosis in several countries worldwide, including Argentina. The immunogen causes only mild clinical signs and does not prevent infection with *A*. *marginale*, but reduces disease severity and prevents death [8]. The absence of anaplasmosis outbreaks in endemic areas is achieved after a high proportion of calves become naturally infected with *A*. *marginale* or by inoculation of young cattle with the *A*. *centrale* live vaccine, along with avoidance of the entry of *A*. *marginale-*infected cattle to anaplasmosis-free zones of Argentina. Implementation of appropriate control measures requires highly sensitive and specific serological tests that could provide mainly information on: i) precise identification of carriers, ii) epidemiological status of anaplasmosis in calves from enzootic regions, and iii) the immune response to *A*. *centrale* vaccine.

Several diagnostic assays have been developed and used in the field, including complement fixation (CF) [9], card agglutination [9,10], indirect fluorescent antibody test (IFAT) [9], dot ELISA [11], indirect enzyme-linked immunosorbent assay (ELISA) [12,13] and competitive ELISA (cELISA) [14]. ELISAs are preferable to conventional tests because of their practicality, objectivity, reliability, suitability for automation, fast turn-around time and often higher sensitivity and specificity for detection of anaplasmosis carriers [4].

The commercial cELISA (ccELISA) recommended by the World Organization for Animal Health (OIE) is based on the *A*. *marginale* recombinant major surface protein 5 (MSP5) fused with *E*. *coli* maltose binding protein (MBP-MSP5) and on the monoclonal antibody (mAb) ANAF16C1. For an endemic area of USA (*A*. *centrale*-free country), 96% sensitivity and 95% specificity were reported using this test [15]. Although ccELISA is used for detecting antibodies against *A*. *centrale*, the test has not been validated for this specific purpose.

MSP5 is a transmembrane protein of 210 residues (23kDa) present in all recognized *Anaplasma* species, and the MSP5 epitope defined by ANAF16C1 is broadly conserved, with cross-reactivity described among *A*. *marginale*, *A*. *centrale*, *A*. *ovis*, *A*. *phagocytophilum* and *Ehrlichia* sp. [16–18]. A strategy to enhance the expression of soluble protein is the use of molecular chaperones. However, this approach could increase nonspecific reactions by interaction of antibodies with chaperones or decrease specific reactions by hiding epitopes due to steric impediments. The chaperone MBP enhances the expression of soluble MSP5, but also increases the number of false positives due to antibodies against *E*. *coli* MBP, which are frequently found in bovines. For this reason, an adsorption step of sera with MBP is required before performing the analysis [14,19]. Chung et al. (2014) evaluated the replacement of MBP with glutathione S-transferase (GST) for the expression of the soluble recombinant antigen in a cELISA to detect antibodies against *A*. *marginale*. The authors reported that three types of problems were solved: i) MBP binders; ii) nonspecific binders of unknown mechanism; and iii) cELISA values close to the cutoff point. However, expression levels, purification or stability of GST-MSP5 were not mentioned [19].

In order to develop a highly specific and sensitive cELISA, not only for the detection of antibodies in cattle infected with *A*. *marginale*, but also in bovines vaccinated with *A*. *centrale*, the test was evaluated with a modified antigen. Two hypothesis were tested in this work: i) the expression of a truncated MSP5 (tMSP5) variant without the amino-terminal hydrophobic region in *E*. *coli* will allow for expression of soluble, recombinant protein avoiding the use of molecular chaperones (MBP or GST); and ii) the inclusion of *A*. *centrale* MSP5 with the *A*. *marginale* antigen as a target in the ELISA will allow for the identification of vaccinated cattle with high sensitivity without affecting the detection of animals naturally infected with *A*. *marginale*.

The aim of this study was to evaluate the use of the MSP5 truncated protein of *A*. *marginale* (tMSP5m), *A*. *centrale* (tMSP5c) and *A*. *centrale-A*. *marginale fusion* (tMSP5cm) as antigen in a cELISA. The sensitivity of the three versions of cELISA developed was analyzed according to the cattle population (naturally infected or previously vaccinated calves).

## Materials and methods

### Cattle samples

Blood samples were aseptically collected with and without 5% citrate as anticoagulant from three cattle groups. The first group included 255 cows born and raised in two Argentinean farms, located in a highly endemic area of anaplasmosis. Both farms, “La Margarita” and “Don Goyo” were located in Avia Terai, (26°40’S—60°46’W), and Villa Angela (27°35’S—60°43’W) respectively, in the Chaco province. The second group included 173 calves vaccinated with a single dose of 10^7^ *A*. *centrale* parasitized erythrocytes, at least 4 months before of the beginning of this study. The utilization of *A*. *centrale* vaccine has been approved by the National Agri Food Health and Quality Service (N° 93159). The calves were from a herd historically free of anaplasmosis, located in a temperate zone free of ticks (Rafaela, 31°11'S—61°30'W), owned by INTA, at the Santa Fe province. The third group included 216 cows, also born and reared in the above-mentioned anaplasmosis-free herd of INTA.

Blood and serum samples obtained from susceptible, healthy and splenectomised cattle infected by intramuscular inoculation of 10^7^ of *A*. *marginale* or *A*. *centrale* were used as positive controls. The cattle were isolated in boxes used *ad hoc* in the Agricultural Experimental Station of the INTA of Rafaela. Food and water was provided *ad libitum* and general health status of the animals was monitored daily. All protocols were approved by the Animal Care Committee of the Faculty of Veterinary Sciences, National University of Litoral (Protocol number 243/15). Samples were distributed in aliquots and kept at -20°C until use.

### DNA extraction

Genomic DNA (gDNA) was obtained from blood samples by the phenol/chloroform method. Briefly, erythrocyte suspensions were lysed with erythrocyte lysis buffer (0.14 M NH_4_Cl, 0.17 M Tris–HCl) at room temperature for 30 min and then pelleted. The hemoglobin was washed off using distilled water by centrifugation at 14,000 × g for 15 min, and pellets were lysed in 400 μl lysis buffer (0.05 M Tris–HCl pH 8.0, 0.1 M EDTA, 0.1 M NaCl, 2% SDS) with 160 μg of proteinase K (Invitrogen Corp., USA) at 58 °C for 1 h. gDNA was extracted with 1 volume of phenol/chloroform/isoamyl alcohol, precipitated with ice-cold isopropyl alcohol and washed once with 75% ice-cold ethanol. Pellets were suspended in 50 μl distilled water and kept at -20 °C until use.

### Status of infection

The status of anaplasmosis infection of sampled cattle was confirmed by nested-PCR (nPCR) specifically validated for each species of *Anaplasma* [20]. Infection of calves vaccinated with *A*. *centrale* was also confirmed by direct microscopic examination of blood smears stained with Giemsa.

### *In silico* analysis of MSP5

Sequence alignment, presentation and determination of percent identity between *A*. *marginale* and *A*. *centrale* MSP5 was performed using CLUSTAL W (1.83) (http://embnet.vital-it.ch/software/ClustalW.html). TMPred algorithm (http://embnet.vital-it.ch/software/TMPRED_form.html) was used to predict the transmembrane domains [21]. Solubility of full-length, truncated MSP5 (residues 28–210) and fusion protein was calculated using a prediction model based on *E*. *coli* overexpressed proteins (http://www.biotech.ou.edu/) [22].

### Cloning of truncated *msp5* gene from *A*. *marginale* and *A*. *centrale*

cDNA encoding residues 28-210 with a six-histidine tag at the C-terminus of the protein MSP5 from *A*. *marginale* (tMSP5m) or MSP5 from *A*. *centrale* (tMSP5c) were amplified by PCR using the primers MSP5-F 5’-catatgggtattttcagcaaaatc-3’ and MSP5-R 5’-ggatcctcagtgatggtgatggtgatggcggccttcaattttaaaagaattaagcatgtgacc-3’. cDNA for tMSP5m and tMSP5c were cloned into pGEM–T Easy (Promega, USA). Subsequently, a fragment was excised with *Nde* I and *Bam*H I and subcloned into pET9b (Novagen, USA) to yield ptMSP5m or ptMSP5c. The identity of the DNA construct was confirmed by sequencing (Instituto de Biotecnología, INTA CICVyA, Argentina).

Three PCR were used to generate the ptMSP5cm: PCR1 by amplification of tMSP5c using ptMSP5c as template and primers 5´-tgcatgcaaggagatggcgcccaacagt-3´ (F-pET9) and 5´-gctttcttatacagagaggtagaattaagcatg-3´ (R-1), PCR2 by amplification of tMSP5m using pMSP5m and the primers 5´-ctctgtataagaaagcaggtggtattttcag-3´(F-2) and 5´-ttctccttcattacagaaacggct-3´ (R-pET9), and PCR3 using PCR1 and PCR2 products as template and primers F-pET9 and R-pET9. Sequence underline of primers R-1 and F-2 are complementary and allow the fusion of the PCR1 and PCR2 products.

### Protein expression and purification

*E*. *coli* BL21 RIL (DE3)*pLysS* competent cells (Novagen, USA) transformed with ptMSP5m, ptMSP5c or ptMSP5cm were cultured at 37°C in 500 ml of Luria–Bertani medium supplemented with 50 μg/ml kanamycin and 34 μg/ml chloramphenicol to *OD*_600 nm_ = 1. Protein expression was induced with 1% lactose. After 3 h of induction at 37°C, bacteria were harvested by centrifugation, suspended in 10 ml of lysis buffer (50 mM sodium phosphate, 300 mM NaCl, 10 mM imidazole, pH 8) containing 1/1000 protease inhibitor cocktail set III (Calbiochem, USA), and lysed by 2 passes through a cell disruptor at 20000 psi (Avestin Emulsiflex B15, Canada). After centrifugation (12000 × g, 30 min, 4°C), the soluble fraction was separated and added to 2 ml of Ni-NTA agarose (Qiagen, Germany) previously equilibrated with lysis buffer. After incubation at 4°C for 1 h, the suspension was poured into a 1.5 cm × 5.0 cm column and washed with 5 volumes of lysis buffer containing 30 mM imidazole. Bound tMSP5m, tMSP5c or tMSP5cm was eluted successively with 5 volumes of 100 mM and then with 5 volumes of 200 mM imidazole lysis buffer. Finally, the buffer was exchanged into 50 mM sodium phosphate, 200 mM NaCl, pH 7.2 by overnight dialysis at 4°C. Molar concentration in pure samples was calculated by absorbance at 280 nm using a molar extinction coefficient (*ε*_280nm_*)* equal to 8940 M^−1^cm^–1^, 10430 M^-1^cm^-1^ or 20860 M^−1^cm^–1^ for tMSP5m, tMSP5c or tMSP5cm, respectively.

### Western blot for tMSP5m, tMSP5c and tMSP5cm

Sodium dodecyl sulfate polyacrylamide gel electrophoresis (SDS-PAGE) was performed as previously described [23]. The proteins were then transferred to nitrocellulose membranes. Free protein-binding sites were blocked by incubation with TBS/5% nonfat milk for 2 h. The mAb ANAF16C1-peroxidase conjugate (VMRD Inc., USA) was evaluated at 1/100 dilution in TBS/0.05% Tween 20/5% nonfat milk. After incubation at 25°C for 1 h, membranes were washed five times with TBS/0.05% Tween 20 and the reaction was revealed by adding the colorimetric substrate 3,3'-diaminobenzidine tetrahydrochloride (DAB) (Sigma-Aldrich, USA).

### ELISA protocols

All field serum samples and controls were assayed in duplicate at room temperature (25°C).

#### Commercial competitive ELISA (ccELISA)

A ccELISA (VMRD Inc, USA) based on the fusion protein MBP-MSP5 recommended by the OIE was used in this study, following the manufacture’s recommendation. Briefly, 70 μl of controls and serum samples were incubated in the MBP adsorption plate at 25°C for 30 min. Then, 50 μl of the adsorbed samples were transferred to the corresponding wells of the *Anaplasma* Antigen-Coated Plate and incubated for 1 h. After two washes with wash solution, 50 μl of mAb ANAF16C1-peroxidase conjugate was added and incubated for 20 min. After washing the plate four times, 50 μl of substrate solution was added to each well and incubated in darkness for 20 min. Finally, 50 μl of stop solution was added and detection was performed at 620 nm.

#### In-house competitive ELISA (hcELISA)

Three versions of hcELISA based on tMSP5m (hcELISA-tMSP5m), tMSP5c (hcELISA-tMSP5c), or tMSP5cm (hcELISA-tMSP5cm) were evaluated. Polystyrene microplates (Thermo Fisher Scientific Inc, USA) were coated overnight at 4°C with 50 μl of purified tMSP5m, tMSP5c, or tMSP5cm (1 μg/well) in PBS. After two washes with PBS, 300 μl of blocking buffer (PBS/10% fat-free dried milk) was added and the plates were incubated for 1 h. The plates were washed three times with PBS/0.05% Tween 20 and then incubated with 100 μl of serum samples for 1 h. Thereafter, the same protocol and reagents of ccELISA were used.

### Data analysis

Results were expressed as percent inhibition (%I), which was calculated using the following formula:

%I=100[1−(SampleOD/NegativeControlOD)]

The optimal cutoff point, sensitivity and specificity for ccELISA and the three versions of hcELISA were established by ROC analysis using the MedCalc 8.1.0.0 software. Concordance between ccELISA and hcELISA-tMSP5m was estimated by Cohen’s kappa value using the same software [24]. Differences among results obtained with the four cELISA protocols were evaluated by a Mann Whitney test.

## Results

### Expression, purification and quality control of recombinant proteins

**Fig 1:**
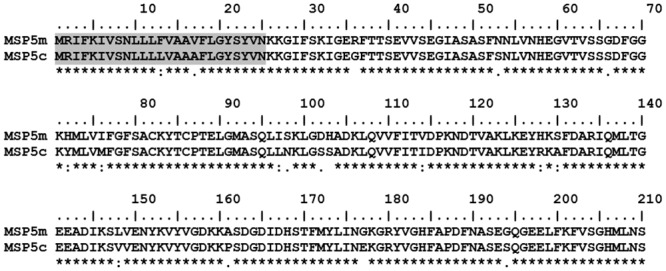
Sequence alignment of *A*. *marginale* MSP5 (MSP5m) and *A*. *centrale* MSP5 (MSP5c). The transmembrane regions, excluded from tMSP5m and tMSP5c, are highlighted with horizontal gray bars. Asterisk (*) indicates positions having a conserved residue; colon (:) indicates conservation among groups of strongly similar characteristics; and period (.) indicates conservation among groups of weakly similar characteristics.

**Fig 2:**
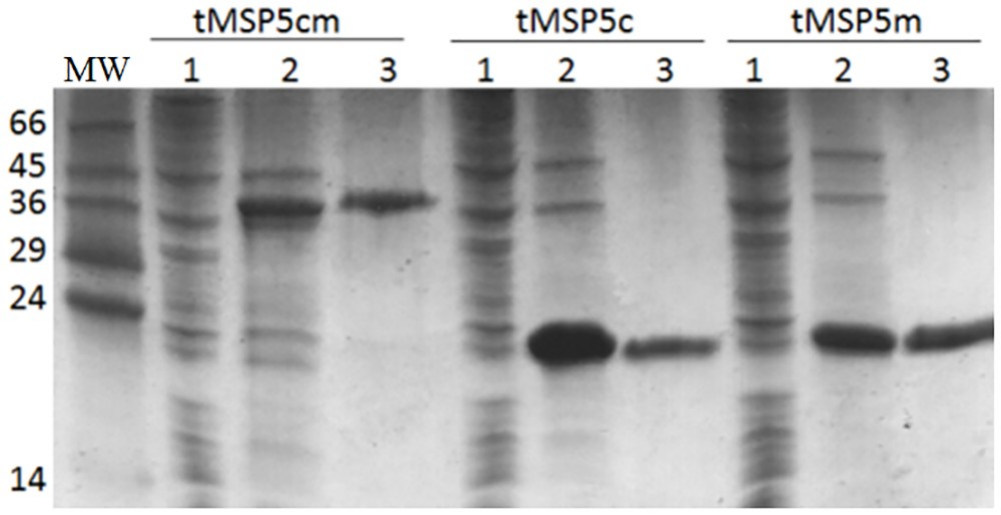
SDS-PAGE of fractions from the purification of tMSP5cm, tMSP5c and tMSP5m on a Ni-NTA column. Lane MW, Molecular weight markers; lanes 1 and 2, post-induction insoluble and soluble fractions of the cell lysate, respectively; lanes 3 corresponds to the purified protein fraction with 100 mM imidazole. The stain was Coomassie Brilliant Blue R-250.

The comparison of the MSP5 protein sequence of *A*. *marginale* and *A*. *centrale* (MSP5m and MSP5c, respectively) revealed 91.5% of identity (Fig 1). The analysis of the primary structure of MSP5 conducted with TMPred predicted a transmembrane helix in the N-terminus of the protein (residues 1–25) [21]. The solubility predicted for MSP5 full-length, truncated (residues 28–210) and fusion proteins, overexpressed in *E*. *coli*, was 65%, 100% and 100%, respectively [22]. MSP5 recombinant proteins (tMSP5m, tMSP5c and tMSP5cm) were expressed with high efficiency in *E*. *coli*, and more than 95% of the recombinant protein was soluble in the bacterial cytoplasm. The amount of expressed protein was approximately 40 mg, 60 mg and 60 mg per liter of culture for tMSP5m, tMSP5c and tMSP5cm, respectively. The C-terminal His tag added to the proteins allowed us to purify large amounts of pure protein (>95%) in a single step (Fig 2).

**Fig 3:**
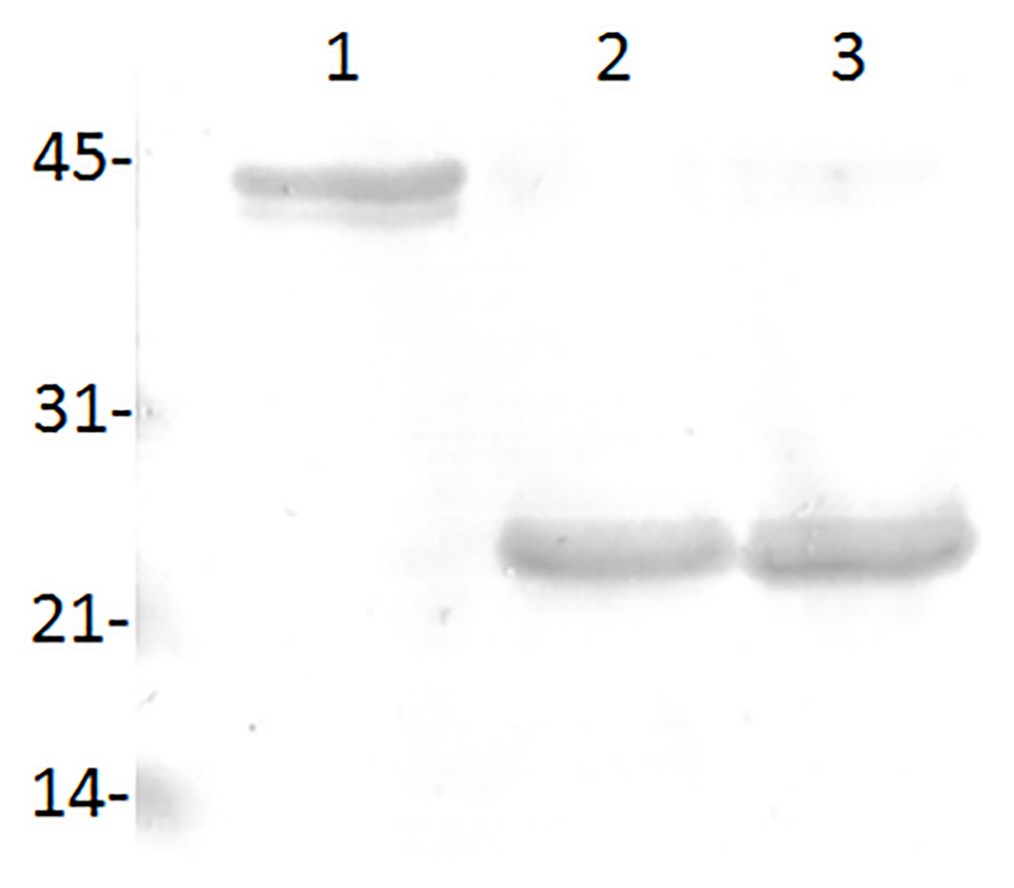
Western blot of purified tMSP5cm (lane 1), tMSP5c (lane 2) and tMSP5m (lane 3) revealed with the monoclonal antibody ANAF16C1.

The immunoreactivity of tMSP5m, tMSP5c and tMSP5cm was demonstrated by western blot with the mAb ANAF16C1, which detected the three recombinant proteins. (Fig 3).

### *Anaplasma* infection status based on nPCR results

*Anaplasma* spp. positive samples were selected and classified using nPCR as gold standard method. Only 226 DNA samples from 255 cows born and raised in a highly endemic area of anaplasmosis were nPCR positive for *A*. *marginale* and negative for *A*. *centrale*. All analyzed vaccinated cattle (n = 173) were positive for *A*. *centrale* and negative for *A*. *marginale*, and all cows born and bred in a farm historically free of anaplasmosis were nPCR negative for both strains (n = 216).

### Antibody detection by ELISA

According to the nPCR results, 226, 173 and 216 serum samples from *A*. *marginale* infected, *A*. *centrale* vaccinated and *A*. *marginale*/*A*. *centrale* negative cattle, respectively, were used for comparing cELISA tests.

**Fig 4:**
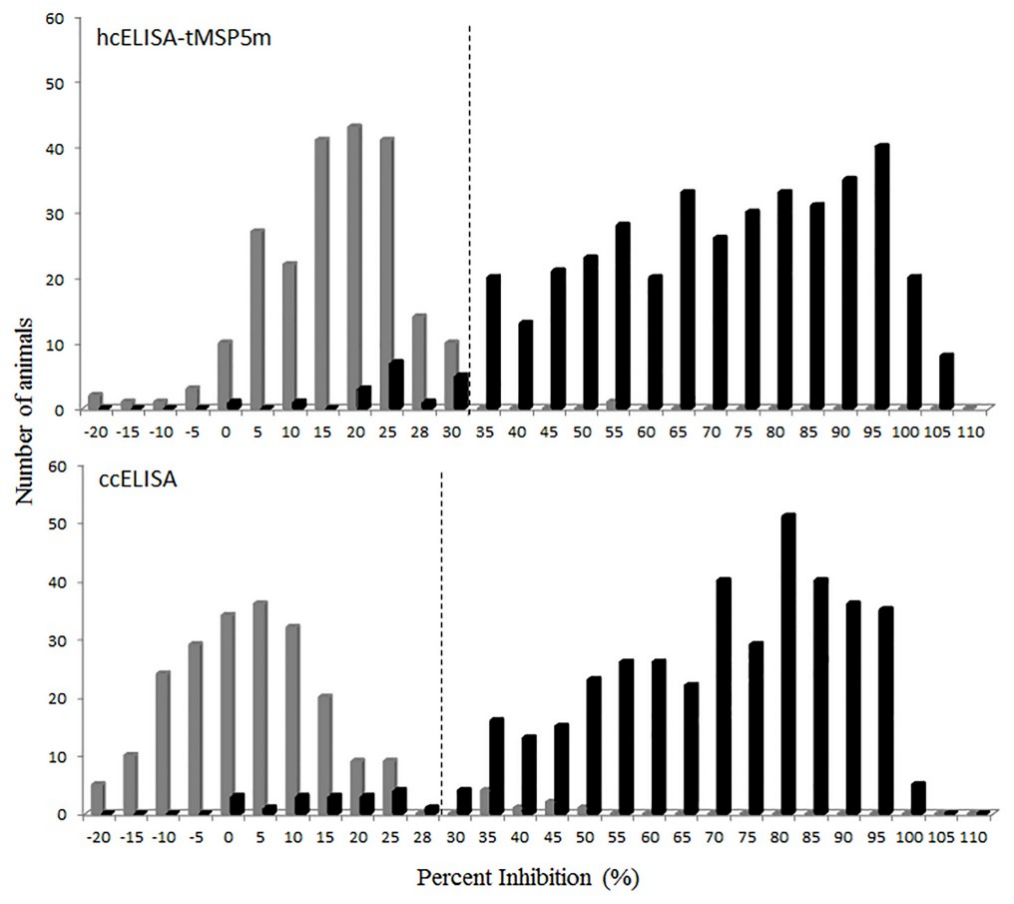
Frequency distribution of hcELISA-tMSP5m and ccELISA values from uninfected (gray bars); and infected or vaccinated (black bars) cattle. Cut-off point (dotted line) was 28 and 30%I for ccELISA and hcELISA-tMSP5m, respectively. Test samples having >28 and >30%I were positive for ccELISA and hcELISA-tMSP5m, respectively.

The frequency distribution of %I for ccELISA and hcELISA-tMSP5m in infected or vaccinated and uninfected bovines is shown in Fig 4. Negative and positive sera had a mean %I of 15 and 68 by hcELISA-tMSP5m and 2 and 66 by ccELISA, respectively. The ROC analysis showed cutoff point values of 30%I with 95.5% of sensitivity and 99.5% of specificity for hcELISA-tMSP5m and 28%I with 95.5% of sensitivity and 96.3% of specificity for ccELISA. The agreement between ccELISA and hcELISA-tMSP5m results was 97% with a *kappa* value of 0.94. A total of 29 false positive and 8 false negative results were detected by hcELISA-MSP5m and/or ccELISA, only 7 of them were coincident by both tests (Table 1).

**Table 1:** False negative and false positive results by ccELISA (MBP-MSP5) vs hcELISA (tMSP5m) for *Anaplasma* spp. antibodies detection.

	Infected (FN)			Uninfected (FP)
	Total (n = 399)	*A*. *marginale* (n = 226)	*A*. *centrale* (n = 173)	n = 216
ccELISA and hcELISA-tMSP5m	7 (1.7%)	4 (1.8%)	3 (1.7)	0 (0.0%)
ccELISA	11 (2.8%)	7 (3.1%)	4 (2.3%)	8 (3.7%)
hcELISA-tMSP5m	11 (2.8%)	2 (0.9%)	9 (5.2%)	1 (0.5%)

Number and proportion of false negative (FN) and false positive (FP) reactions in sera from cattle infected with *Anaplasma marginale* or vaccinated with *A*. *centrale* (n = 399) and uninfected cattle (n = 216).

### Comparison between ccELISA and hcELISA

**Fig 5:**
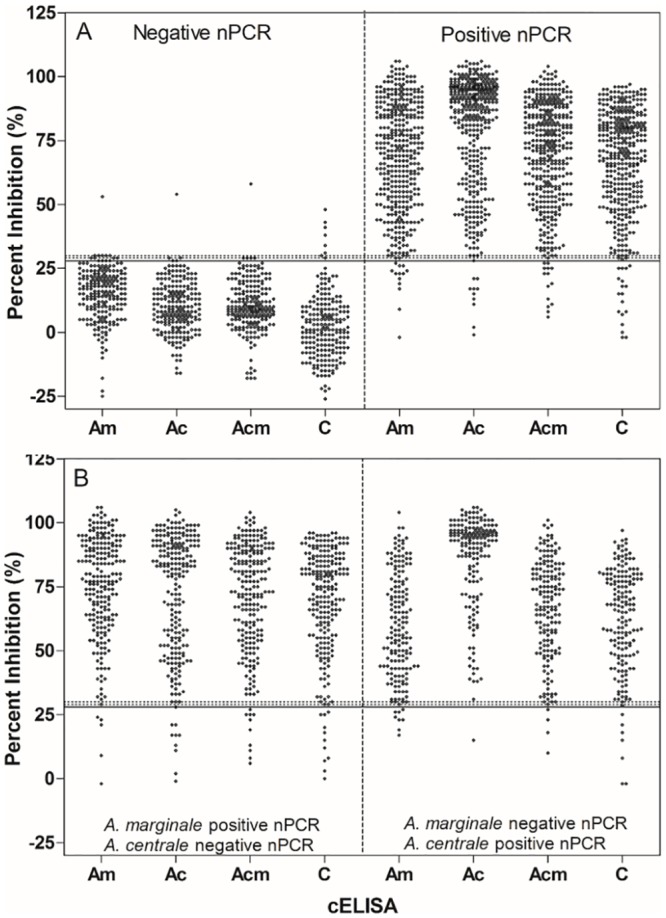
Percent inhibition results obtained by hcELISA-tMSP5m (Am), -tMSP5c (Ac), -tMSP5cm (Acm) and ccELISA (C). A) *Anaplasma* spp. nPCR negative and positive cattle, and B) *A*. *marginale-*infected (left) and *A*. *centrale-*vaccinated (right) cattle. The cut-off values for ccELISA and hcELISA are indicated with a solid and dotted line, respectively.

The hcELISA based on the truncated MSP5 variants tMSP5m, tMSP5c or tMSP5cm showed higher sensitivity and specificity than the ccELISA based on MBP-MSP5 antigen. Specificity was the same for the three versions (99.5%) and sensitivity was 95.5%, 96.2% and 97.2% for hcELISA-tMSP5m, hcELISA-tMSP5cm and hcELISA-tMSP5c, respectively. The best performance was observed with hcELISA-tMSP5c and the highest difference was obtained when infected and vaccinated cattle were analyzed separately (Table 2). Percent inhibition as measured by ccELISA and hcELISA in infected and uninfected cattle are shown in Fig 5A; those levels for cattle infected with *A*. *marginale* or vaccinated with *A*. *centrale* are shown in Fig 5B. For the negative population, the median of %I values of hcELISA-tMSP5c and hcELISA-tMSP5cm were similar and statistically different from the hcELISA-tMSP5m and ccELISA (p<0.0001). For the positive population, the median of %I values of hcELISA-tMSP5m, hcELISA-tMSP5cm and ccELISA were similar and statistically different from the hcELISA-tMSP5c (<0.0001) (Table 3).

**Table 2:** Sensitivity and specificity of commercial cELISA (ccELISA) and three versions of *in-house* cELISA (hcELISA) development for serological diagnosis of cattle infected with *Anaplasma marginale* or vaccinated with *A*. *centrale*.

Antigen (cut-off point)	hcELISA			ccELISA
	tMSP5m (%I > 30)	tMSP5c (%I > 29)	tMSP5cm (%I > 29)	MBP-MSP5 (%I > 28)
Specificity (%)	99.5	99.5	99.5	96.3
Sensitivity (%)	95.5	97.2	96.2	95.5
Sensitivity (%)	97.3	95.6	95.6	95.1
Sensitivity (%)	93.1	99.4	97.1	96.0

%I: percent inhibition.

**Table 3:** Median and interquartile range of percent inhibition (% I) calculated for each of the three versions of hcELISA and ccELISA to detect antibodies in bovines naturally infected with *A*. *marginale*, vaccinated with *A*. *centrale* and in non-infected cattle.

	hcELISA (%I)			ccELISA (%I)
Antigen	tMSP5m	tMSP5c	tMSP5cm	MBP-MSP5
Negative cattle (n = 216)	16 (9–22)^b^	9 (4–17)^a^	9 (5–17)^a^	1(-7-9)^c^
Positive cattle (n = 399)	71 (52–87)^a^	87 (61–95)^b^	72 (54–85)^a^	70 (53–82)^a^
Infected (n = 226)	77 (62–91)^a^	82 (52–92)^a^	75 (56–89)^a^	74 (56–85)^a^
Vaccinated (n = 173)	57 (43–77)^a^	93 (78–97)^b^	69 (52–81)^a^	66 (48–79)^a^

Between brackets: interquartile range. Within the same cattle group, different superscript letters following values indicate statistical significance (p<0.0001).

## Discussion

In this work, truncated MSP5 from *A*. *marginale* and *A*. *centrale* was expressed without a chaperone protein, with high yield, solubility and purity, without losing the immunoreactivity. The replacement of MBP-MSP5 of *A*. *marginale* in the ccELISA with either of the three variants of the truncated MSP5 of *A*. *marginale* (tMSP5m) or *A*. *centrale* (tMSP5c) or both together as fusion protein (tMSP5cm) improved the performance of hcELISA, particularly by increasing the specificity, which was achieved by eliminating cross-reactions against MBP.

ccELISA is currently used in Argentina to identify cattle herds with enzootic instability for anaplasmosis, which should be protected by the *A*. *centrale* live vaccine, and to assess the immune response after vaccination [13,25,26]. *A*. *marginale* MSP5 has shown distinctive attributes to be considered the protein of choice for the diagnosis of anaplasmosis. MSP5 is encoded by a single copy gene, conserved in *A*. *marginale* isolates of different geographical origins, and shows high identity with MSP5 from other species of *Anaplasma* [16–18]. Knowles et al. (1996) developed a cELISA based on the MBP-MSP5 fusion protein and the mAb ANAF16C1, which was validated using sera from *A*. *marginale* naturally or experimentally infected cattle [14]. This test has also been used to detect antibodies against *A*. *centrale*; however its actual performance has not been studied in this context. Despite its usefulness, the test has some disadvantages inherent to the fusion protein that affects specificity [14,19].

Overexpression of the full-length MSP5 protein in *E*. *coli* produces insoluble inclusion bodies. High-level expression of many recombinant proteins in *E*. *coli* leads to the formation of highly aggregated proteins commonly referred to as inclusion bodies. The solubility of recombinant antigens is a requirement for developing a serological diagnostic test. For this purpose, the protein aggregates must be solubilized and refolded, with the additional inconvenience that only a small fraction of the initial protein is recovered [27]. Several reagents (new vectors, host strains) and strategies (chaperone co-expression, low temperature induction, composition of the culture media) have been developed to optimize the soluble expression of recombinant proteins in *E*. *coli* [28]. Knowles et al. (1996) avoided precipitation of MSP5 in inclusion bodies by the co-expression with the MBP chaperone molecule, which boosted solubility and refolding, improving the yield of the soluble recombinant protein [14]. MBP has shown to be more efficient to solubilize the partner proteins than GST and thioredoxin (Trx), two other commonly used chaperon proteins [29]. However, MBP is involved in the maltose/maltodextrin system of *E*. *coli* and interferes with the diagnosis test [19]. This ubiquitous bacterium is often the cause of enteric and mammary infections in bovines, and prompts antibodies against MBP. Therefore, the sera analyzed by cELISA based on MBP-MSP5 require previous adsorption with MBP to avoid false positive reactions [14]. The sensitivity and specificity reported for ccELISA in *A*. *marginale* naturally infected cattle varied according to the gold standard used for bovine classification, with sensitivity ranging from 65.2% to 99.2% and specificity from 83.3% to 99.5% [10,15,30].

The *in silico* analysis showed that the most favorable condition to express the soluble recombinant MSP5 protein was without its transmembrane region. Cloning and expression of recombinant proteins without the transmembrane region may drastically increase the expression levels and solubility of heterologous proteins expressed in *E*. *coli* [31]. Replacing MBP-MSP5 fusion protein in ccELISA with truncated MSP5 proteins (tMSP5m, tMSP5c or tMSP5cm) reduced the running time and increased the test sensitivity and specificity for detecting antibodies against both *A*. *marginale* and *A*. *centrale*. The novel antigens fulfilled the requirements for determining the epidemiological status of *Anaplasma* spp. in the endemic area where discrimination between these species is not required.

In this work, the expression of the truncated MSP5 without N-terminus transmembrane helix (residues 1–27) of *A*. *marginale* (tMSP5m), *A*. *centrale* (tMSP5c) or fusion protein (tMSP5cm) was obtained in the soluble fraction with high purity in a single step and did not form aggregates during its conservation. Chung et al. (2014) expressed GST-MSP5 in *E*. *coli* by solubilizing the transmembrane helix in detergent micelles and the crude soluble fraction was used in a ccELISA for detecting antibodies against *A*. *marginale*. Replacement of MBP-MSP5 with GST-MSP5 showed higher specificity, comparable sensitivity and improved resolution of the ccELISA [19]. However, yield, stability and facility of production for the fusion protein were not reported; the efficacy of this test to identify cattle vaccinated with *A*. *centrale* was not reported either. The ccELISA was developed in the United States, where *A*. *centrale* is not present. In Argentina, as well as in other countries, the vaccine strain of *A*. *centrale* is used in cohorts of calves up to 10 months old in areas with enzootic instability and in calves from the anaplasmosis-free zones which will be moved to an endemic region [32–34].

A cELISA based on crude *A*. *centrale* antigen was able to discriminate *A*. *marginale* in naturally infected cattle from *A*. *centrale* in vaccinated cattle with 97.3% or 98.6% of sensitivity for beef and dairy cattle, respectively, and 98.8% of specificity for both. However, crude antigens are difficult to standardize and there is no commercial test available for that purpose [35].

The novel antigens, which lack a chaperone protein, have allowed us to simplify the ccELISA (MBP-MSP5 antigen) protocol, avoiding the sera adsorption step used to eliminate most of false positive reactions. The three versions of hcELISA showed better performance than ccELISA in cattle infected with *A*. *marginale* as well as in those immunized with *A*. *centrale*. The high specificity (99.5%) observed for hcELISA-tMSP5m, -tMSP5c and tMSP5mc, revealed the elimination of cross-reactions detected by ccELISA, even after serum adsorption with MBP.

The hcELISA showed identical (hcELISA-tMSP5m) or higher (hcELISA-tMSP5c and–tMSP5cm) sensitivity than that detected by ccELISA. Interestingly, the highest sensitivity was detected by hcELISA-MSP5c, followed by hcELISA-tMSP5cm, both including MSP5 of *A*. *centrale*. When sera from infected and vaccinated cattle were evaluated separately, the sensitivity and the median values of %I (hcELISAs) were enhanced when homologous antigens were used. The use of the fusion protein (tMSP5cm) increased the overall sensitivity by improving the detection level in the vaccinated group.

In conclusion, the truncated MSP5 protein allowed an increase in the specificity of the cELISA to 99.5% and the generation of a fusion protein among *A*. *centrale* and *A*. *marginale* tMSP5 protein allowed an increase in the cELISA sensitivity when vaccinated animals were analyzed.

## References

[1] Theiler A. *Anaplasma marginale* (Gen. and spec. nov.) The marginal points in the blood of cattle suffering from a specific disease. Rep Gov Veeterinary Bacteriol Transvaal Dep Agric South Africa 1908–1909. 1910;7–64.

[2] Aubry P, Geale DW. A review of bovine anaplasmosis. Transbound Emerg Dis. 2011;58(1):1–30. 10.1111/j.1865-1682.2010.01173.x 21040509

[3] Kocan KM, De La Fuente J, Blouin EF, Coetzee JF, Ewing SA. The natural history of *Anaplasma marginale*. Vet Parasitol. 2010;167(2–4):95–107. 10.1016/j.vetpar.2009.09.012 19811876

[4] OIE. Manual of Diagnostic Tests and Vaccines for Terestrial Animals. Bovine Anaplasmosis 2015, Chapter 2.4.1.

[5] De Echaide ST, Bono F, Farber M, Lugaresi C, Mangold A, Aguirre N, et al. Anaplasmosis bovina en rebaños lecheros de la provincia de Santa Fe, Argentina. 2004.

[6] Anziani O. Anaplasmosis en áreas libres de garrapatas. In: Memoria de la Reunión Anual de Información técnica. Inst Nac Tecnol Agropecu Estac Exp Reg Agropecu Rafaela. 1979;(63–68).

[7] Eriks IS, Stiller D, Palmer GH. Impact of persistent *Anaplasma marginale* rickettsemia on tick infection and transmission. J Clin Microbiol. 1993;31(8):2091–6. 8370734 10.1128/jcm.31.8.2091-2096.1993 PMC265702

[8] Abdala A, Pipano E, Aguirre D, Gaido A, Zurbriggen M, Mangold A, et al Frozen and fresh *Anaplasma centrale* vaccines in the protection of cattle against *Anaplasma marginale* infection. Rev Elev Med Vet Pays Trop. 1990;43(2):155–8. 2092348

[9] Gonzalez E, Long R, Todorovic R. Comparisons of the Complement-Fixation, Indirect Fluorescent Antibody, and Card AgglutinationTests for the Diagnosis of Bovine Anaplasmosis. Am J Vet Res. 1978;39:1538–41. 358874

[10] Molloy JB, Bowles PM, Knowles DP, Bock RE, Kingston TG, Blight GW, et al Comparison of a competitive inhibition ELISA and the card agglutination test for detection of antibodies to *Anaplasma marginale* and *Anaplasma centrale* in cattle. Aust Vet J. 1999;77(4):245–9. 10330556 10.1111/j.1751-0813.1999.tb11712.x

[11] Montenegro-James S, Guillen A, Ma S, Tapang P, Abdel-Gawad A, Toro M, et al Use of the dot enzyme-linked immunosorbent assay with isolated *Anaplasma marginale* initial bodies for serodiagnosis of anaplasmosis in cattle. Am J Vet Res. 1990;51(10):1518–21. 2240769

[12] Duzgun A, Schuntner C, Wright L, Leatch G, Waltisbuhl D. A Sensitive ELISA Technique for the Diagnosis of *Anaplasma marginale* Infections. Vet Parasitol. 1988;29:1–7. 3176298 10.1016/0304-4017(88)90002-7

[13] De Echaide ST, Bono MF, Lugaresi C, Aguirre N, Mangold A, Moretta R, et al Detection of antibodies against *Anaplasma marginale* in milk using a recombinant MSP5 indirect ELISA. Vet Microbiol. 2005;106(3–4):287–92. 10.1016/j.vetmic.2004.12.026 15778035

[14] Knowles D, De Echaide ST, Palmer G, McGuire T, Stiller D, McElwain T. Antibody against an *Anaplasma marginale* MSP5 Epitope Common to Tick and Erythrocyte Stages Identifies Persistently Infected Cattle. J Clin Microbiol. 1996;34(9):2225–30. 8862589 10.1128/jcm.34.9.2225-2230.1996 PMC229221

[15] De Echaide ST, Knowles DP, McGuire TC, Palmer GH, Suarez CE, McElwain TF. Detection of cattle naturally infected with *Anaplasma marginale* in a region of endemicity by nested PCR and a competitive enzyme-linked immunosorbent assay using recombinant major surface protein 5. J Clin Microbiol. 1998 3;36(3):777–82. 9508311 10.1128/jcm.36.3.777-782.1998 PMC104624

[16] Strik NI, Alleman AR, Barbet AF, Sorenson HL, Wamsley HL, Gaschen FP, et al Characterization of *Anaplasma phagocytophilum* major surface protein 5 and the extent of its cross-reactivity with *A*. *marginale*. Clin Vaccine Immunol. 2007;14(3):262–8. 10.1128/CVI.00320-06 17215333 PMC1828860

[17] Visser ES, McGuire TC, Palmer GH, Davis WC, Shkap V, Pipano E, et al The *Anaplasma marginale* msp5 gene encodes a 19-kilodalton protein conserved in all recognized Anaplasma species. Infect Immun. 1992;60(12):5139–44. 1280624 10.1128/iai.60.12.5139-5144.1992 PMC258289

[18] Al-Adhami B, Scandrett WB, Lobanov VA, Gajadhar AA. Serological cross-reactivity between *Anaplasma marginale* and an *Ehrlichia* species in naturally and experimentally infected cattle. J Vet Diagn Invest. 2011;23(6):1181–8. 10.1177/1040638711425593 22362799

[19] Chung C, Wilson C, Bandaranayaka-Mudiyanselage CB, Kang E, Adams DS, Kappmeyer LS, et al Improved diagnostic performance of a commercial *Anaplasma* antibody competitive enzyme-linked immunosorbent assay using recombinant major surface protein 5-glutathione S-transferase fusion protein as antigen. J Vet Diagn Invest. 2014;1;26(1):61–71. 10.1177/1040638713511813 24318928

[20] Molad T, Mazuz ML, Fleiderovitz L, Fish L, Savitsky I, Krigel Y, et al Molecular and serological detection of *A*. *centrale*- and *A*. *marginale*-infected cattle grazing within an endemic area. Vet Microbiol. 2006;113(1–2):55–62. 10.1016/j.vetmic.2005.10.026 16300909

[21] Hofmann K, Stoffel W. A Database of Membrane Spanning Protein Segments. Biol Chem. 1993;374:166.

[22] Diaz AA, Tomba E, Lennarson R, Richard R, Bagajewicz MJ, Harrison RG. Prediction of protein solubility in *Escherichia coli* using logistic regression. Biotechnol Bioeng. 2010;105(2):374–83. 10.1002/bit.22537 19739095

[23] Schägger H, von Jagow G. Tricine-sodium dodecyl sulfate-polyacrylamide gel electrophoresis for the separation of proteins in the range from 1 to 100 kDa. Anal Biochem. 1987;166(2):368–79. 2449095 10.1016/0003-2697(87)90587-2

[24] Metz C. Basic principles of ROC analysis. Semin Nucl Med. 1978;10;8(4):283–98. 112681 10.1016/s0001-2998(78)80014-2

[25] Palacios C, De Echaide ST, Mattion. Evaluation of the immune response to *Anaplasma marginale* MSP5 protein using a HSV-1 amplicon vector system or recombinant protein. Res Vet Sci. 2014;97(3):514–20. 10.1016/j.rvsc.2014.10.005 25458492

[26] Mastropaolo M, De Echaide ST, Cuatrín A, Arece H, Lobato S&, Mangold AJ. Situación de la babesiosis y anaplasmosis de los bovinos en el sudoeste de la provincia del Chaco. FAVE. 2009;9(1):29–35.

[27] Singh SM, Panda AK. Solubilization and refolding of bacterial inclusion body proteins. J Biosci Bioeng. 2005;99(4):303–10. 10.1263/jbb.99.303 16233795

[28] Francis DM, Page R. Strategies to optimize protein expression in E. coli. Curr Protoc Protein Sci. 2010;(SUPPL. 61):1–29. 20814932 10.1002/0471140864.ps0524s61 PMC7162232

[29] Kapust RB, Waugh DS. *Escherichia coli* maltose-binding protein is uncommonly effective at promoting the solubility of polypeptides to which it is fused. Protein Sci. 1999;8(8):1668–74. 10.1110/ps.8.8.1668 10452611 PMC2144417

[30] Dreher U, de la Fuente J, Hofmann-Lehmann R, Meli M, Pusterla N, Kocan K, et al Seroprevalence of anaplasmosis among cattle in Switzerland in 1998 and 2003: no evidence of an emerging disease. Clin Diagn Lab Immunol. 2005;12(10):1177–83. 10.1128/CDLI.12.10.1177-1183.2005 16210480 PMC1247822

[31] Yang Z, Ahn H-J, Nam H-W. High expression of water-soluble recombinant antigenic domains of *Toxoplasma gondii* secretory organelles. Korean J Parasitol. 2014;52(4):367–76. 10.3347/kjp.2014.52.4.367 25246715 PMC4170032

[32] Anziani O, Tarabla H, Ford C, Galleto C. Vaccination with *Anaplasma centrale*: response after an experimental challenge with *Anaplasma marginale*. Trop Anim Heal Prod. 1987;19(2):83–7. 10.1007/BF02297324 3629722

[33] Potgieter F, Van Rensburg L. Infectivity virulence and immunogenicity of *Anaplasma centrale* live blood vaccine. Onderstepoort J Vet Res. 1983;50(1):29–31. 6348636

[34] Potgieter F. Epizootiology and control of anaplasmosis in south africa. J S Afr Vet Assoc. 1979;50(4):367–72. 399978

[35] Molloy JB, Bock RE, Templeton JM, Bruyeres AG, Bowles PM. Identification of antigenic differences that discriminate between cattle vaccinated with Anaplasma centrale and cattle naturally infected with Anaplasma marginale. Int. J. Parasitol. 2001;31:179–86. 11239938 10.1016/s0020-7519(00)00145-4

